# Allosteric Regulation of HIV-1 Reverse Transcriptase by ATP for Nucleotide Selection

**DOI:** 10.1371/journal.pone.0008867

**Published:** 2010-01-25

**Authors:** Masaru Yokoyama, Hiromi Mori, Hironori Sato

**Affiliations:** Pathogen Genomics Center, National Institute of Infectious Diseases, Musashi Murayama-shi, Tokyo, Japan; Institut Pasteur, France

## Abstract

**Background:**

Human immunodeficiency virus type 1 reverse transcriptase (HIV-1 RT) is a DNA polymerase that converts viral RNA genomes into proviral DNAs. How HIV-1 RT regulates nucleotide selectivity is a central issue for genetics and the nucleoside analog RT inhibitor (NRTI) resistance of HIV-1.

**Methodology/Principal Findings:**

Here we show that an ATP molecule at physiological concentrations acts as an allosteric regulator of HIV-1 RT to decrease the *K_m_* value of the substrate, decrease the *k_cat_* value, and increase the *K_i_* value of NRTIs for RT. Computer-assisted structural analyses and mutagenesis studies suggested the positions of the ATP molecule and NRTI-resistance mutations during a catalytic reaction, which immediately predict possible influences on nucleotide insertion into the catalytic site, the DNA polymerization, and the excision reaction.

**Conclusions/Significance:**

These data imply that the ATP molecule and NRTI mutations can modulate nucleotide selectivity by altering the fidelity of the geometric selection of nucleotides and the probability of an excision reaction.

## Introduction

Human immunodeficiency virus type 1 reverse transcriptase (HIV-1 RT) is an RNA-dependent DNA polymerase that converts single-stranded viral RNA genomes into double-stranded proviral DNAs after HIV-1 entry into the cells. Active HIV-1 RT is composed of two related chains, termed p51 and p66 [1]. The p66 chain has a catalytic site for DNA polymerization: the fingers, palm, and thumb subdomains form a cavity for the binding of the template, primer, two divalent cations, and dNTPs for DNA synthesis [1], as seen in other DNA polymerases. Although HIV-1 RT exhibits no exonucleolytic proofreading activity, it still retains a relatively high level of fidelity of DNA synthesis, i.e., about 2.5–6×10^−4^ base substitutions per site [2], [3]. Increasing evidence suggests that the high fidelity of DNA synthesis achieved by DNA polymerases—i.e., the discrimination of the correct and incorrect nucleotides for polymerization—is primarily due to the geometric selection of nucleotides during nucleotide insertion into the catalytic site [4], [5], [6].

An ATP molecule is a multifunctional nucleotide that exists at a concentration of ∼3.2 mM in the cells [7]. Many studies have suggested that the ATP molecule is a cellular factor involved in the drug resistance of HIV-1. Nucleoside analog RT inhibitors (NRTIs) act as chain terminators blocking DNA synthesis, since they lack the 3′-OH group required for the phosphodiester bond formation, whereas NRTI-resistant RT catalyzes dinucleoside polyphosphate synthesis in the presence of millimolar concentrations of NTP [8]. Thus, the ATP molecule at physiological concentrations *in vitro* serves as an effective pyrophosphate donor to the excision reaction of the RT to remove the chain terminating NRTIs [8], [9], [10]. A previous crystal structure study identified a binding site of ATP in the catalytic cavity of p66 when the RT was free from the template and primer [11]. Although ATP-mediated excision provides a plausible mechanism for the NRTI resistance of HIV-1, some NRTI-resistance mutations are located distantly from the excision site. Therefore, their roles in NRTI resistance are not fully understood [12].

Enzyme activity is often modulated by an allosteric effector, a small natural compound that binds to the enzyme at a site distinct from the substrate-binding site. In this study, we show by kinetic, structural, and mutagenesis studies that the ATP molecule can act as an allosteric effector of HIV-1 RT to modulate nucleotide selectivity and DNA polymerization. We also show probable three-dimensional (3-D) positions of the bound ATP molecule and NRTI-resistance mutations during a catalytic cycle. The obtained data suggest that the ATP molecule and NRTI mutations can cooperatively modulate physicochemical properties of the p66 catalytic cavity to alter the fidelity of the geometric selection of nucleotides and the probability of an excision reaction.

## Results

### Effects of ATP on HIV-1 RT Reaction Kinetics

First, we analyzed the effects of ATP on HIV-1 RT reaction kinetics. We began by collecting basic information on the steady-state kinetics of DNA polymerization in the absence of the ATP molecule. We used two HIV-1 RTs for the present study: the NRTI-sensitive RT (93JP-NH1) and multi-NRTI-resistant RT (ERT-mt6) [13]. The ERT-mt6 RT has an 11-amino-acid insertion in the β3-β4 loops of the p66 fingers subdomain and four substitutions–M41L, T69I, L210W, and T215Y–in the polypeptide backbone of 93JP-NH1 [13]. These mutations confer higher levels of resistance of the 93JP-NH1 virus against AZT, d4T, β-L-2′,3′-dideoxy-3-thiacytidine, 2′,3′-dideoxyinosine, and 2′,3′-dideoxycytidine than other mutants in the polypeptide backbone of 93JP-NH1 [13]. Therefore, we used ERT-mt6 RT, which clearly showed that NRTI-resistance mutations enhance the effect of ATP on enzyme kinetics in NRTI-sensitive RT.

The initial velocities of dTTP incorporation into poly (rA)·p(dT)_12-18_ were measured using purified p51/p66 RT heterodimers of the 93JP-NH1 and ERT-mt6 RTs (Figure S1A). In both RTs, the dTTP incorporation followed Michaelis-Menten kinetics (Figure S1B). The *K_m_* values for the 93JP-NH1 and ERT-mt6 RTs were 4.0±0.1 and 13.8±0.5 µM, respectively, suggesting that the dTTP has a higher *K_m_* value for ERT-mt6 RT. The *k_cat_* values for 93JP-NH1 and ERT-mt6 RTs were 1.04±0.01 and 0.49±0.01s^−1^, respectively, suggesting that ERT-mt6 RT has a nucleotide addition reaction with a slower turnover rate.

**Figure 1:**
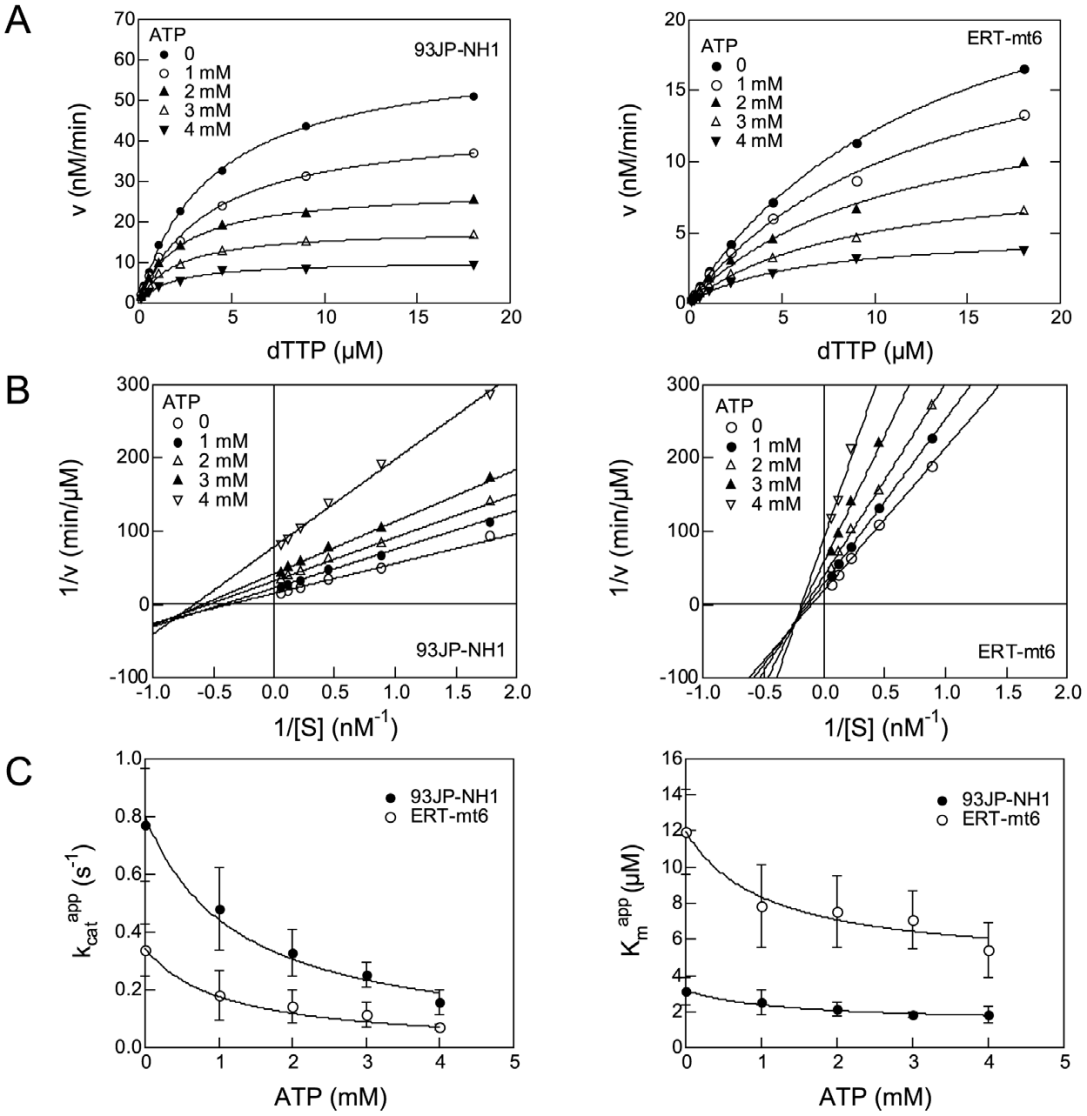
Effects of ATP on HIV-1 RT reaction kinetics. **A.** The substrate-velocity curves of purified HIV-1 RTs in the presence of ATP. RNA-dependent DNA polymerase activity [13] of the purified RTs was measured using various concentrations of [α-^32^P]dTTP and poly (rA)·p(dT)_12-18_ in the presence of ATP. Representative results with 93JP-NH1 RT (left) and ERT-mt6 RT (right) are shown. **B.** Lineweaver-Burk double-reciprocal plots for ATP-dependent inhibition of dTTP incorporation. Reciprocal values of the initial velocities and substrate concentrations in Figure 1A are plotted. **C.** Effects of ATP on *K_m_* (left) and *k_cat_* (right) values in RT reaction. The *K_m_* and *k_cat_* values were estimated by fitting of the initial velocity of dTTP incorporation to Equations 3 and 4 as described in Materials and Methods. The mean values with variances of the six independent experiments are shown.

We then used the two RTs to examine whether or not the ATP molecule influences the DNA polymerization kinetics by measuring the initial velocity of dTTP incorporation into poly (rA)·p(dT)_12-18_ in the presence of 0, 1, 2, 3, and 4 mM of ATP. The velocity is decreased in both RTs in association with an increase in ATP concentration, indicating that the ATP molecule inhibits the overall catalytic reaction by HIV-1 RT (Figure 1A). Lineweaver-Burk double-reciprocal plots showed that, in both RTs, the straight lines at the different ATP concentrations have different x- and y-intercepts, and that the line slope increases with increasing ATP concentration (Figure 1B). The data suggest that the ATP-mediated inhibition of dTTP incorporation is a mixed noncompetitive inhibition.

We further examined whether or not the ATP molecule can influence the *K_m_* of substrate and *k_cat_* values in the enzyme reaction. The substrate-velocity data in Figure 1A were fit to Equations 1 and 2 (see Materials and Methods), and the average *K_m_* and *k_cat_* values in the presence of 0, 1, 2, 3, and 4 mM of ATP were obtained with six independent experiments. Notably, both *K_m_* and *k_cat_* values for the 93JP-NH1 and ERT-mt6 RTs monotonically decreased with increasing ATP concentration (Figure 1C).

We also examined the *K_i_* values of ATP to the two RTs. On the basis of information on the RT catalytic cycle [14], we assumed two structures of RT for ATP binding: the RT-template-primer complex (RT complex 1) and the RT-template-primer-dTTP complex (RT complex 2). Using Equations 3 and 4 (see Materials and Methods), we calculated *K_i_^ATP^* and *K_i_′ ^ATP^* values of ATP to the RT complexes 1 and 2, respectively. The *K_i_^ATP^* values were 2.9±1.3 and 2.8±1.3 mM for the 93JP-NH1 and ERT-mt6 RTs, respectively, suggesting that the ATP molecule binds with the equivalent *K_i_^ATP^* value to complex 1 of the two RTs. The *K_i_′ ^ATP^* values were 1.2±0.5 and 1.1±0.4 mM for the 93JP-NH1 and ERT-mt6 RTs, respectively, suggesting that the ATP molecule also binds with the equivalent *K_i_′ ^ATP^* value to complex 2 of these RTs. These results are consistent with the finding that AZT resistance mutations cause no difference in ATP binding [15], [16]. Finally, the *K_i_^ATP^* value was larger than the *K_i_′ ^ATP^* value in both RTs, suggesting that ATP binds with higher affinity to the dTTP-bound RT than to the substrate-free RT.

### ATP's Effects on NRTI Action

**Figure 2:**
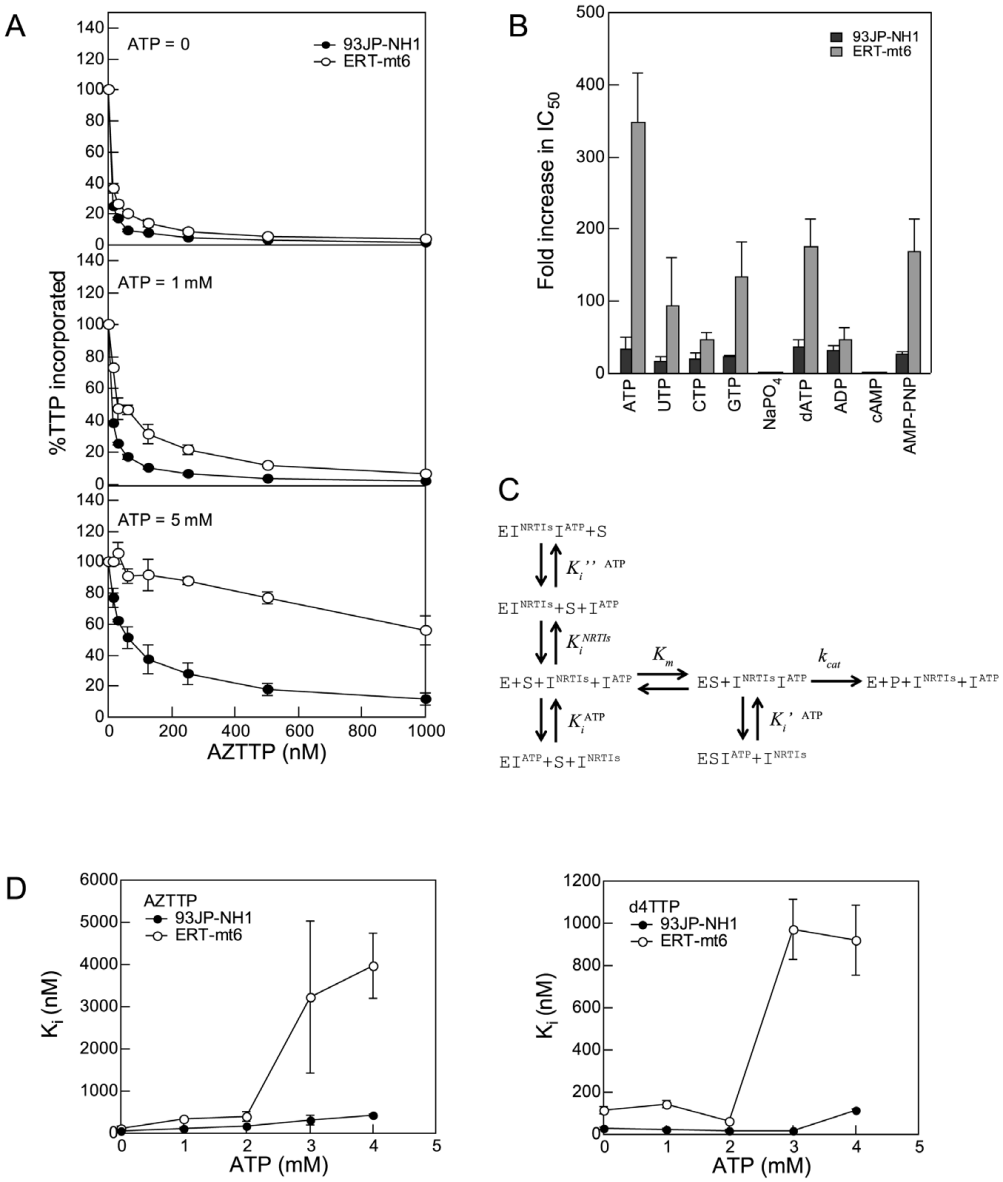
Effects of ATP on NRTI action. **A.** Effects of ATP on AZTTP-dependent inhibition of RT activities. The dTMP incorporation into poly (rA)·p(dT)_12-18_ was measured using [α-^32^P]dTTP and purified p51/p66 heterodimers in the presence of the indicated concentrations of ATP and AZTTP. Ratios of the dTTP incorporation at given concentrations of AZTTP to that in the absence of AZTTP are shown. **B.** Effects of nucleotides and related compounds on IC_50_s of AZTTP. IC_50_s of AZTTP were determined in the presence of 5 mM of the indicated compounds, and the fold increases in IC_50_ compared to AZTTP without the compounds are shown. **C.** A simplified kinetics model of DNA polymerization in the presence of ATP and NRTI. The model was generated on the basis of the kinetics data in Figure 1 and Figure S2, previously reported kinetics data [17], [18], and a crystal structure study of the ATP-RT complex [11]. **D.** Effects of ATP on the *K_i_* values of AZTTP and d4TTP. The *K_i_^AZTTP^* and *K_i_^d4TTP^* values were estimated by fitting the initial velocity of dTTP incorporation to Equation 5 as described in Materials and Methods. The mean values with variances are shown for two independent experiments performed with duplicate samples.

Next, we examined whether or not the ATP molecule influences the action of NRTI on the two RTs. In the absence of the ATP molecule, nanomole orders of AZTTP effectively inhibited dTTP incorporation by both RTs (Figure 2A, top), suggesting that, in the absence of the ATP molecule, the level of inhibition of DNA polymerization by AZTTP is equivalent between the two RTs. In the presence of 1 mM ATP, the inhibition was more moderate with the ERT-mt6 RT than with the 93JP-NH1 RT, and the difference in the inhibition curve became much greater in the presence of 5 mM ATP (Figure 2A, middle and bottom). These data suggest that the ATP molecule and NRTI mutations cooperatively reduce the NRTI sensitivity of HIV-1 RT *in vitro*.

We next examined whether or not the above ATP effects on NRTI resistance were specific to the ATP molecule by measuring the IC_50_ of AZTTP in the presence of 5 mM UTP, CTP, GTP, NaPO_4_, dATP, ADP, cAMP, and AMP-PNP (Figure 2B). While many of the compounds tested increased the IC_50_ of AZTTP, the magnitude of the fold increase was consistently greater with ERT-mt6 RT than 93JP-NH1 RT. The ATP molecule was most effective at increasing the IC_50_, yielding an approximately 350-fold increase with the ERT-mt6 RT. The ATP, dATP, and GTP molecules, which have a purine ring, had greater effects on the IC_50_ increase than the UTP and CTP molecules, which have a pyrimidine ring. NaPO_4_ and cAMP had little effect on the IC_50_ in either RT. Notably, AMP-PNP, a non-hydrolyzed analogue of the ATP molecule, also increased the IC_50_ of AZTTP by approximately 150-fold with the ERT-mt6 RT. These data suggest that nucleotides similar in size to the ATP molecule can assist in the development of NRTI resistance.

We further examined whether or not the ATP molecule influences the *K_i_* values of AZTTP and d4TTP to the two RTs in the enzyme reaction. Based on the kinetics data for ATP in Figure 1, the kinetics data for AZTTP in Figure S2, the reported kinetics data for AZTTP inhibition [17], [18], and a crystal structure study of the ATP-RT complex [11], a simplified kinetics model in which ATP functions as a mixed noncompetitive inhibitor and nucleoside analogs function as competitive inhibitors was hypothesized (Figure 2C). We measured the initial velocities of dTTP incorporation by HIV-1 RTs at various ATP concentrations and various AZTTP or d4TTP concentrations, and calculated the *K_i_* values of these compounds for the 93JP-NH1 and ERT-mt6 RTs using Equations 5 and 6 (see Materials and Methods). Notably, the ATP molecule induced increases in the *K_i_^NRTI^* value in a dose-dependent manner. The magnitudes of the increases were much greater with ERT-mt6 RT, reaching about 30-fold for the *K_i_^AZTTP^* value and 8.5-fold for the *K_i_^d4TTP^* value at 3 mM of ATP compared to those without the ATP molecule (Figure 2D). The magnitude of changes in *K_i_^NRTI^* values was much smaller with 93JP-NH1 RT, which yielded about 6.8- and 0.6-fold increases in *K_i_^AZTTP^* and *K_i_^d4TTP^* values, respectively, at 3 mM of ATP compared to those without the ATP molecule. These data suggest that physiological concentrations of ATP [7] potently increase the *K_i_* value of NRTI when RT has NRTI-resistance mutations.

### Structural Study on ATP Action

**Figure 3:**
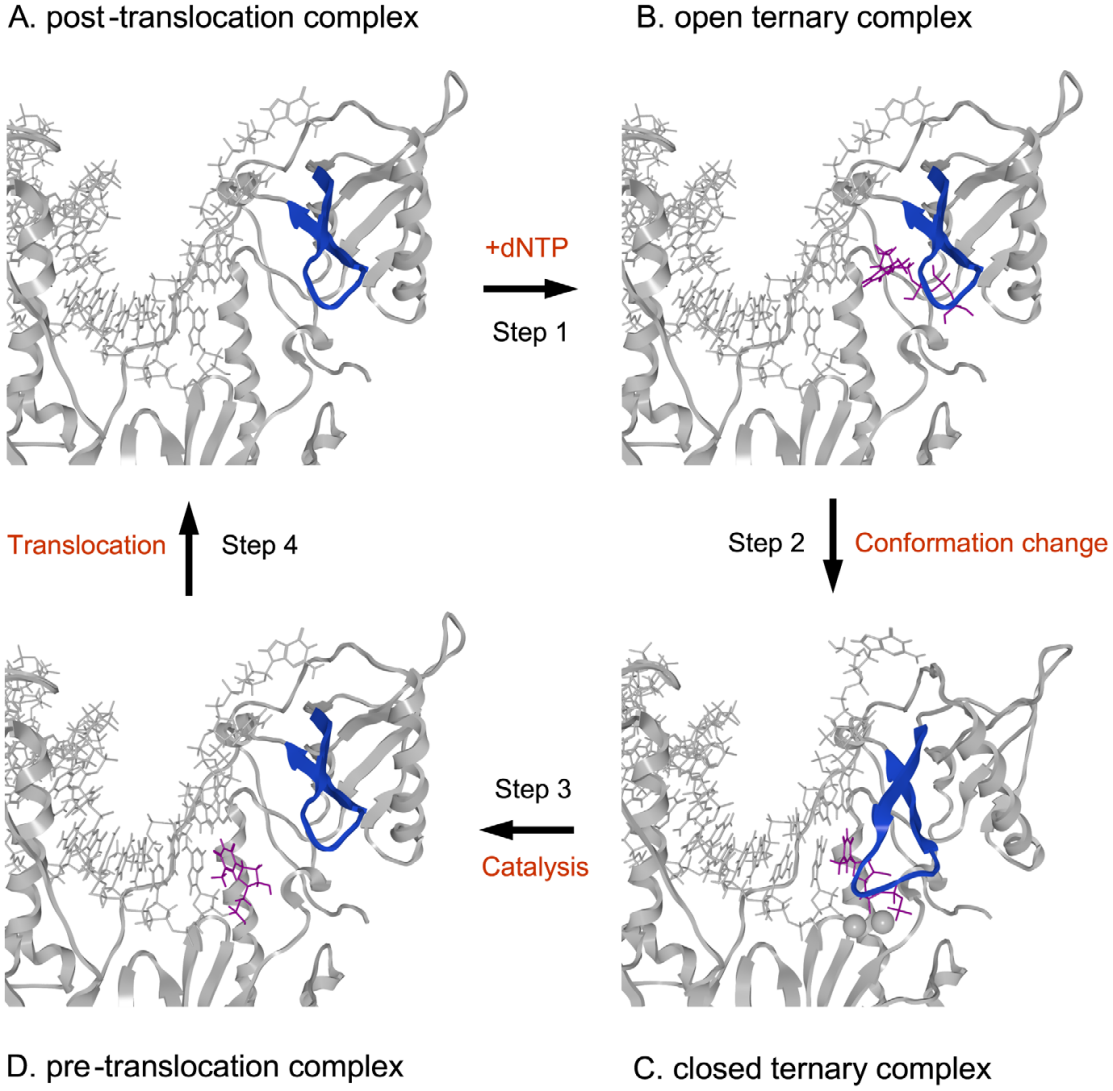
Structural models of the HIV-1 RT p66 subunit in a DNA polymerization cycle. The 3-D models of the 93JP-NH1 p66-template-primer ternary complex of the fingers-open configuration at post-translocation (**A**), fingers-open configuration at the stage of dTTP binding (**B**), fingers-closed configuration after fingers-rotation (**C**), and fingers-open configuration at pre-translocation stage (**D**). The models were constructed by homology modeling and docking simulation techniques using two crystal structures [1], [14] of the HIV-1 RTs as modeling templates (see Materials and Methods). Catalytic clefts composed of fingers, palm, and thumb subdomains are shown. dTTP, magenta sticks; p66 main chain, grey ribbon; Mg^2+^ ion, gray spheres; template-primer, grey sticks; β3-β4 loop of the fingers subdomain, blue ribbon.

A previous study identified a binding site of the ATP molecule in the p66 when the RT was free from the template and primer [11]. To address the binding site of the ATP molecule in RT during DNA synthesis, we conducted a computer-assisted structural analysis. Using the homology modeling method [19], we first constructed 3-D models of 93JP-NH1 and ERT-mt6 RTs at various catalytic stages defined by biochemical and crystallographic data [14], [20], [21] (Figure 3). In the DNA polymerization processes, each single nucleotide addition cycle was divided into four steps, termed the post-translocation, fingers-open ternary, fingers-closed ternary, and pre-translocation complex stages [14] (Figure 3).

The 3-D models were used to search for a possible binding site for nucleotides by docking simulations [22]. A previous study suggested that incoming dNTP and NRTIs bind along the p66 fingers subdomain of the fingers-open ternary RT complex at the post-translocation stage [23] (Figures 3A and 3B), as is generally seen in other polymerases [24], [25], [26], [27], [28]. The binding position was in agreement with the biochemical mode of NRTI inhibition, competitive inhibition, as shown by our kinetic study of AZTTP (Figure S2) and previous studies [17], [18]. This position was also consistent with the position needed to initiate the base pair formation at the enzyme active center after rotation (∼20°) of the β3-β4 loops to form a fingers-closed ternary complex [5] (Figures 3B and 3C).

**Figure 4:**
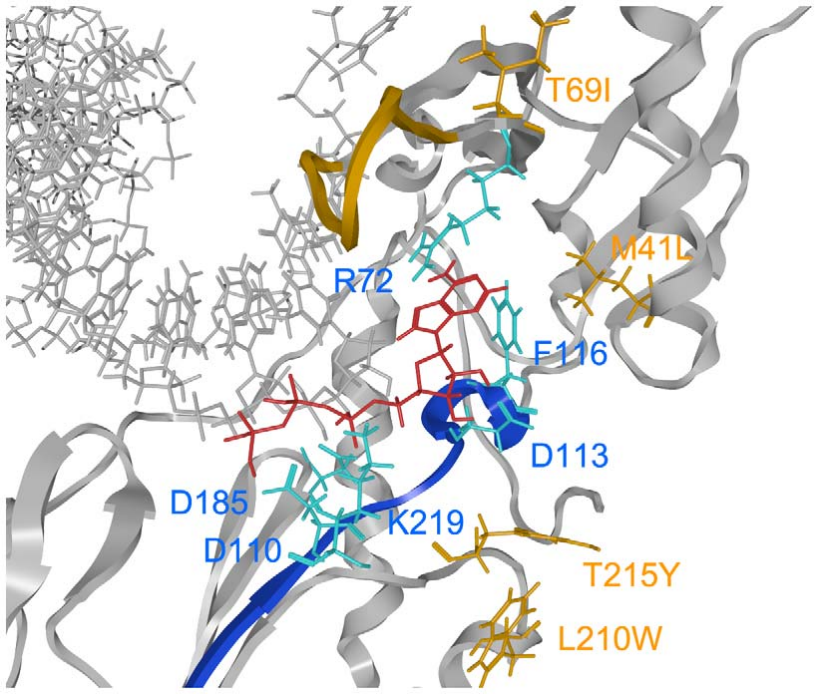
Docking simulations of ATP to the HIV-1 RT p66 subunit with NRTI resistance. ATP was docked with the optimized p66-template-primer complex of the ERT-mt6 strain at the pre-translation stage, using the automated ligand docking program ASEDock2005 [22] operated in the Molecular Operating Environment (see Materials and Methods). Catalytic clefts composed of fingers, palm, and thumb subdomains are shown. ATP, red sticks; p66 main chain, grey ribbon; template-primer, grey sticks; motif A, blue ribbon. The side chains of amino acids around ATP are indicated with cyan sticks, and the side chains of amino acids for NRTI resistance (M41L, T69I, L210W, and T215W) with orange sticks. The main chain of an 11-amino-acid insertion at the β3-β4 loops for NRTI resistance is shown in orange.

The 3-D models of pre- and post-translocation complexes corresponding to E and ES in the kinetic model (Figure 2C) were used to search for a possible binding site for the ATP molecule by docking simulations [22]. The simulations suggested that the ATP molecule could bind to the 93JP-NH1 and ERT-mt6 RTs at the pre-translocation (Figure 3D) and the post-translocation (Figure 3A) stages. The ATP molecule was predicted to bind along the highly conserved motif A near the side chains of R72, D110, D113, F116, D185, and K219 residues at the p66 fingers subdomain of both RTs (Figure 4). The ATP-binding position was stabilized through electrostatic and hydrophobic interactions between the ATP molecule and the side chains of surrounding amino acids. The ATP position was similar to the ATP position in the crystal structure of the template-primer-free RT [11] and was indistinguishable between the pre- and post-translocation stages (Figure S3), suggesting that a specific ATP-binding site is preserved in the free-RT and RT-template-primer tertiary complex. The ATP-binding position was distinct from that of dNTP at initial binding [23] and after fingers-domain rotation [1] in the fingers-open and -closed configurations of RT, respectively (Figures 3 and 4), consistent with our kinetic data for allosteric regulation (Figures 1 and 2).

The bound ATP molecule was located near the YMDD motif, motif A, and the 3′-end of the primer, suggesting that the ATP binding can modulate polymerization and support the excision reaction (Figure 4). The bound ATP molecule was positioned between the catalytic site and the β3-β4 loops, suggesting that the ATP binding can modulate the initial binding and translocation of dNTP and NRTIs into the catalytic site (Figures 3 and 4). Taken together, these structural data are well consistent with our kinetic data, biochemical data for excision [8], [9], [10], and crystal structure data for ATP binding [11].

NRTI-resistance mutations of the ERT-mt6 p66 (Figure 4, orange residues) were located relatively far away from the bound ATP molecule and catalytic center in p66. Thus, it is less likely that these mutations directly influence the ATP-mediated excision. Instead, the M41L, T69I, L210W, and T215Y substitutions augmented the hydrophobicity of the catalytic cavity of p66, which could enhance p66's ability to exclude water from the catalytic site cleft for a higher fidelity of nucleotide selection [6], [29]. This possibility is consistent with our kinetic data. The fingers-domain insertion induced changes in the shape of the β3-β4 loops that could alter the position of the initial binding site of dNTP and NRTI relative to the catalytic site.

### Site-Directed Mutagenesis Study

We further examined how substitutions of amino acids around the predicted ATP-binding site would influence the biochemical properties of the ERT-mt6 RT. Single-amino-acid substitutions were introduced into the p66 chain of ERT-mt6, and their effects on the overall DNA polymerization activity, IC_50_ of AZTTP, *K_m_* value, *k_cat_* value, and *K_i_* value of ATP for the ERT-mt6 RT were analyzed. The positions of the substitutions introduced corresponded to positions 72, 110, 113, 116, and 219 of the 93JP-NH1 p66. We did not conduct mutagenesis of D185 in the YMDD loops, because its essential role in the translocation of the template primer has been established [14]. All of the tested substitutions changed the overall DNA polymerization activity of the RT (Figure S4B). The substitutions at positions 72, 110, and 116 (R72A/Q, D110A/N, and F116A/L) resulted in a loss of DNA polymerization activity, suggesting their essential role in this activity. In contrast, those at positions 113 and 219 (D113A/N and K219Q/A) enhanced the incorporation of dTTP (Figure S4B), suggesting their regulatory role in overall DNA polymerization activity.

**Figure 5:**
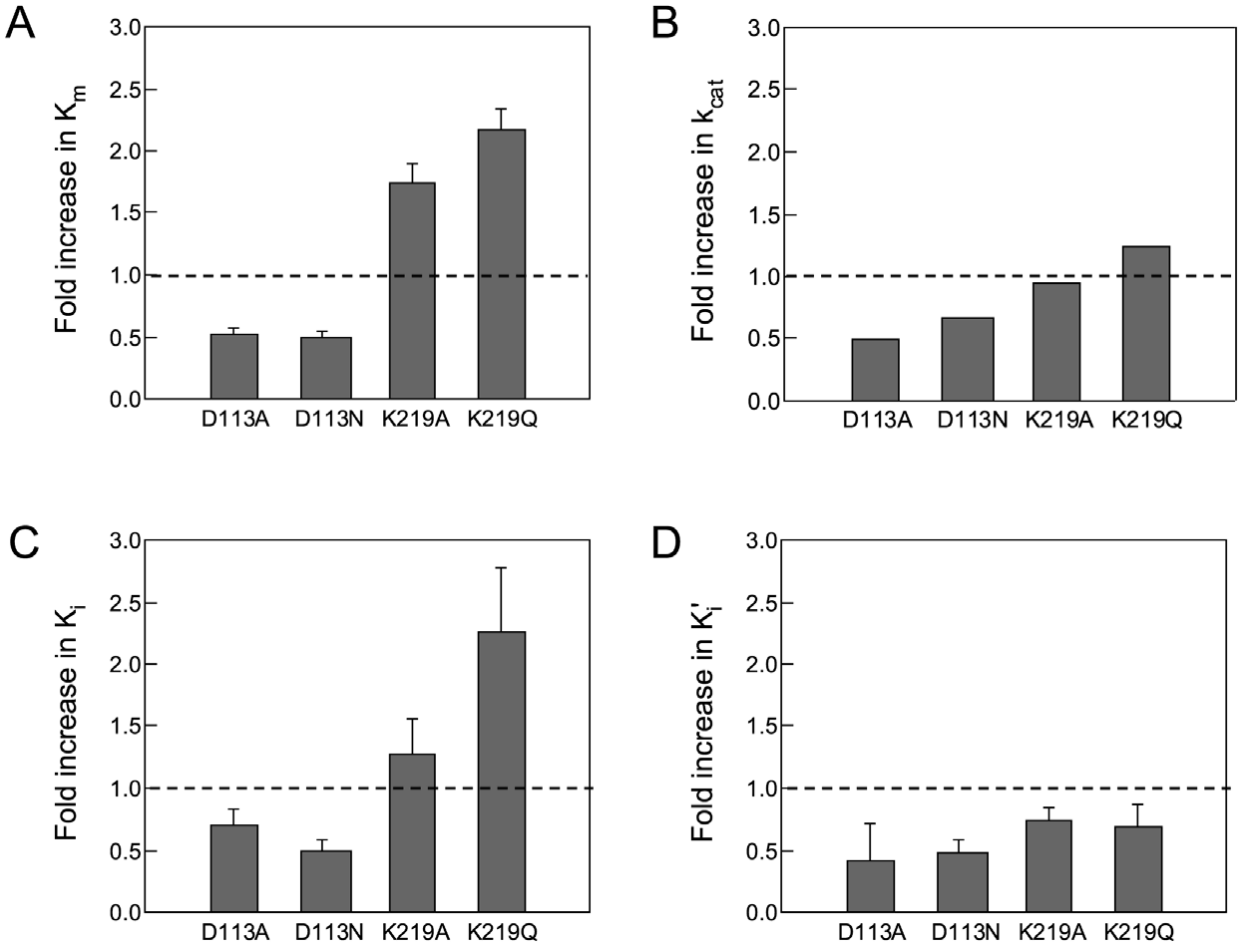
Site-directed mutagenesis study of the HIV-1 RT p66 subunit with NRTI resistance. Single substitutions of amino acids around the predicted ATP-binding site in Figure 3 were introduced into the p66 chain of ERT-mt6. The overall DNA polymerization activity (Figure S4B), IC_50_ of AZTTP (Figure S4C), and *K_m_*, *k_cat_*, *K_i_^ATP^*, and *K_i_′ ^ATP^* values were measured using the [α-^32^P]dTTP and poly (rA)·p(dT)_12-18_ system, and fold increases in the *K_m_* (**A**), *k_cat_* (**B**), *K_i_^ATP^* (**C**), and *K_i_′ ^ATP^* values (**D**) compared to those for the ERT-mt6 were calculated. Results for the RT mutants, D113A, D113N, K219Q, and K219A, which retained sufficient polymerization activity for a kinetic study, are shown.

The active D113A/N and K219Q/A RTs were further examined for changes in the IC_50_ of AZTTP, and in the *K_m_*, *k_cat_*, *K_i_^ATP^*, and *K_i_′ ^ATP^* values. The D113A/N resulted in an approximately 4- to 5-fold reduction in the IC_50_ of AZTTP (Figure S4C), suggesting that D113 plays an important role in the development of NRTI resistance. *K_m_*, *k_cat_*, *K_i_^ATP^*, and *K_i_′ ^ATP^* values were estimated by using the substrate-velocity curves for the D113A/N and K219Q/A RTs (Figure S4D). The D113A/N and K219Q/A substitutions induced changes in the *K_m_*, *k_cat_*, *K_i_^ATP^*, and *K_i_′ ^ATP^* values, suggesting that the D113 and K219 residues regulate the *K_m_* of substrate, *k_ca_*_t_, and *K_i_* values of ATP (Figure 5). The D113A/N substitutions resulted in reductions in *K_m_* and *k_cat_* values, which paralleled the reductions in *K_i_^ATP^* and *K_i_′ ^ATP^* values. The K219Q/A substitutions resulted in increases in *K_m_* values, which paralleled the increases in *K_i_^ATP^* values. The kinetics data implied that residues 113 and 219 can regulate the affinity of the substrate and ATP molecule but do not contribute directly to the catalysis of DNA polymerization, suggesting that the ATP binding site would be distinct from the catalytic site for DNA polymerization.

## Discussion

How HIV-1 RT regulates the nucleotide selectivity for DNA synthesis is a central issue for genetics and the NRTI resistance of HIV-1. In this study, we showed that the ATP molecule at physiological concentrations acted as an allosteric regulator of HIV-1 RT to modulate nucleotide selectivity. We also showed probable 3-D positions of the bound ATP molecule and NRTI mutations in the catalytic cleft; these positions immediately suggested that a nucleotide-selection mechanism—i.e., an ATP- and RT-mutation-mediated modulation of the geometric selection of nucleotides—played a role in the DNA polymerization and NRTI resistance of HIV-1.

First, we demonstrated that the ATP molecule modulated the *K_m_* and *k_cat_* values of the substrate for HIV-1 RT. We showed that the ATP molecule reduced the *K_m_* values of dTTP with both NRTI-sensitive and -resistant RTs (Figure 1C). These results suggested that the ATP molecule can decrease the *K_m_* value of a natural substrate to HIV-1 RT. We also showed that the ATP molecule reduced the *k_cat_* values of these RTs (Figure 1C). These results suggested that the ATP molecule can decrease the rate of DNA polymerization and thereby increase the probability of an excision reaction by HIV-1 RT. Lineweaver-Burk double-reciprocal plots showed that the ATP molecule is a mixed noncompetitive inhibitor of RT, suggesting distinct binding sites for ATP and dNTP. Taken together, these data strongly suggest that the ATP molecule can act as an allosteric regulator to modulate the nucleotide selectivity of HIV-1 RT.

We next investigated the mechanisms by which the ATP molecule modulates the nucleotide selectivity of HIV-1 RT. Docking simulations predicted that the ATP-binding site would be similar between NRTI-sensitive and -resistant RTs during DNA polymerization. Consistent with the results of the kinetic study, the predicted ATP-binding site was distinct from that of dNTP and NRTIs [23], and single-amino-acid substitutions at positions 113 and 219 around the predicted ATP-binding site indeed induced significant changes in the *K_i_* value of ATP. Importantly, the ATP-binding position suggested possible mechanisms by which ATP could influence DNA polymerization and the excision reactions of RT, as follows: First, interactions between the γ-phosphate of the ATP molecule and the side chain of D185 in the YMDD loops could influence the DNA translocation of the primer template [14]. Second, interactions between charged portions of the ATP molecule and the side chains of D110 and D185 could modulate the Mg^2+^ position and stability for DNA polymerization [1]. Third, the γ-phosphate of the ATP molecule is located near the 5′ phosphate of DNA primer terminus and thus could increase the probability of a DNA excision [11] in concert with a reduction in the *k_cat_* value of RT.

We found no marked increases in the *K_i_* values of AZTTP and d4TTP at the ATP concentrations around the *K_i_′ ^ATP^* value (1.1±0.4 mM) for ATP binding to the RT-template-primer-dTTP complex for the excision reaction (Figures 2C and 2D). The ATP molecule was estimated to bind with equivalent *K_i_* values to the NRTI-sensitive and -resistant RTs, as others have indicated [15], [16], suggesting that NRTI-resistance mutations do not necessarily increase ATP-binding affinity. Moreover, some NRTI-resistant mutations, such as M41L and T69I, are located relatively distantly from the ATP-binding site and catalytic site (Figure 4), which makes their direct impact on DNA excision unlikely. Thus, our study suggests that an ATP-mediated DNA excision mechanism alone is insufficient to explain the roles of the NRTI-resistance mutations, as was also noted previously [12]. Therefore, we speculate that NRTI-resistance mutations can decrease the affinity of NRTIs to HIV-1 RT in concert with the ATP molecule.

Our data imply that the ATP molecule and NRTI mutations can modulate the nucleotide selectivity of HIV-1 RT by influencing the geometric selection of nucleotides in the catalytic cavity, as follows: First, the presence of bound ATP molecule in the catalytic cavity can sterically influence the initial binding and translocation of dNTP/NRTI into the catalytic site. Second, more hydrophobic side chains of the NRTI-resistance mutations can improve p66's ability to exclude water from the catalytic cavity and allow more intimate interactions between nucleotides, the primer-template, and amino acids around the catalytic site for distinguishing between correct and incorrect base pairs. In this regard, previous crystal structure analyses have revealed that the active site of a low-fidelity polymerase is more accessible to the solvent than those of more accurate polymerases [30], [31]. Third, *K_i_* ^NRTIs^ values increased sharply with NRTI-resistant RT (ERT-mt6 RT) at ATP concentrations around the *K_i_^ATP^* value (2.8±1.3 mM) for ATP binding to the RT-template-primer complex (Figures 2C and 2D). Fourth, the changes in the *K_m_* value of dTTP correlated with those in the *K_i_* value of the ATP molecule to the RT-template-primer complex (Figures 5A and 5C). Taken together, our structural, kinetic and mutagenesis data suggest that the NRTI-resistance mutations and the ATP molecule can cooperatively modulate physicochemical properties of the p66 catalytic cavity to alter the fidelity of the geometric selection of nucleotides and the probability of an excision reaction.

In conclusion, we demonstrated that the ATP molecule at physiological concentrations acts as an allosteric regulator of HIV-1 RT to decrease the *K_m_* value of the substrate, decrease the *k_ca_* value*_t_*, and increase the *K_i_* value of NRTIs for RT. The effects were independent of NRTI-resistance mutations of RT. The ATP molecule and NRTI mutations could decrease RT's sensitivity to NRTI of RT in concert with the RT mutation. Our data support the notion that the ATP molecule and NRTI mutations can modulate nucleotide selectivity by altering the fidelity of the geometric selection of nucleotides and the probability of an excision reaction.

## Materials and Methods

### Nucleotides

Poly(rA)•p(dT)_12-18_, dNTPs (100 mM, pH 7.5), NTPs (100 mM, pH 7.5), and [α-^32^P]dTTP were purchased from Pharmacia Biotech Inc. (USA). ADP and AMP were from ICN (USA). Adenosine 5′-(β, γ-imido) triphosphate (AMP-PNP) was from Sigma Chemical (USA). 3′-Azido 3′-deoxythymidine 5′-triphosphate (AZTTP), 3′-deoxy-2′, and 3′-didehydrothymidine 5′-triphosphate (d4TTP) were from Moravek Biochemicals (USA).

### Expression and Purification of HIV-1 RT

HIV-1 infectious molecular clones, 93JP-NH1 and ERT-mt6 [13], were used to clone and express the p51 and p66 subunits of the HIV-1 RT. The open reading frames encoding the RT p51 and p66 subunits of the 93JP-NH1 and ERT-mt6 RTs were amplified by PCR and cloned into the BamH1 site of pQE-9 (Qiagen, Germany). The nucleotide sequences of the PCR-amplified fragments and the sequences around the cloning sites were verified with an automated sequencer. Each subunit was expressed individually in XL1-blue by induction with isopropyl-β-D-thiogalactopyranoside, and the cells expressing p51 and p66 were mixed in binding buffer (20 mM sodium phosphate, 500 mM NaCl, 10 mM imidazole, and EDTA-free protease inhibitor mixture (Roche, Germany), lysed with a French press, centrifuged at 10,000 g for 20 min, and filtered (0.45-µm pore size). The p51/p66 heterodimers were purified from the filtered lysates by Ni^2+^ affinity chromatography (HiTrap Chelating HP; Amersham Biosciences, UK) and size exclusion chromatography (HiLoad 16/60 Superdex 200 pg; Amersham Biosciences, UK). All of the purification processes were carried out at 4°C. About 1.5 (ERT-mt6) and 3 mg (93JP-NH1) of the p51/p66 heterodimers with greater than 95% purity as judged by SDS-polyacrylamide gel electrophoresis (Figure S1) were obtained with a 1-liter culture. The specific activities of the purified RTs were 40,000 and 10,000 units/mg of protein for 93JP-NH1 and ERT-mt6, respectively, wherein one unit is defined as the amount of enzyme required for incorporation of 1.0 nmol of ^32^P-dTTP into poly(rA)/poly(dT)_12-18_ in 10 min at 37°C.

### Measurement of RT Activity

The purified RTs were dissolved in the RT stock buffer (50 mM Tris-HCl pH 7.5, 75 mM KCl, 5 mM MgCl_2_, 2 mM DTT, 0.05% NP40, and 50% glycerol)[32], [33] and kept at −30°C until use. RNA-dependent DNA polymerase activity was measured using [α-^32^P]dTTP and poly(rA)/poly(dT)_12-18_ as described previously [33]. For the RT reaction in the presence of ATP, RT activities were measured in 100 µl of RT reaction cocktail consisting of 50 mM Tris-HCl pH 7.5, 75 mM KCl, 5 mM MgCl_2_, 2 mM DTT, 0.05% NP40, and 50% glycerol containing RT (1–10 nM), dTTP (0.2–18 µM), and ATP (0–4 mM). For the RT reaction in the presence of ATP and NRTI, RT activities were measured in 100 µl of the RT reaction cocktail containing RT (1–10 nM), dTTP (0.2–18 µM), ATP (0–5 mM), and AZTTP or d4TTP (0–1 µM). These experiments were performed in duplicate and repeated two to six times.

### Steady-State Kinetic Analysis

The averages of the experimental data were fit by a nonlinear regression method using the program Igor Pro (WaveMetrics, USA). The kinetics parameters were determined by the Michaelis-Menten equation:
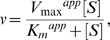
(1)where [*S*] is the substrate concentration; *K_m_^app^* is the apparent Michaelis-Menten constant; and *V_max_^app^* is the apparent maximal rate attained when the enzyme active sites are saturated by substrate.

Based on the kinetics data in Figure 1 and Figure S2, the previously reported kinetics data [17], [18], and a crystal structure study of the ATP-RT complex [11], we assumed mixed noncompetitive inhibition of ATP and competitive inhibition of NRTIs (Figure 2C).

Using this model, the enzyme kinetic parameters were calculated using Equations 2–5.

*V_max_^app^* and *K_m_^app^* are defined as
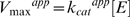
(2)and
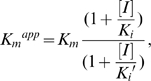
(3)respectively, where *K_m_* is the Michaelis-Menten constant; [*E*] is the enzyme concentration; [*I*] is the inhibitor concentration; *K_i_* and *K_i_′* are the inhibition constants for the enzyme and the complex of the enzyme with substrate; and *k_cat_^app^* is the apparent turnover number:
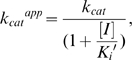
(4)where *k_cat_* is the turnover number.

The *k_cat_^app^* and *K_m_^app^* that can be derived from the model (Figure 2C) are
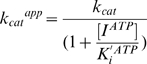
(5)and
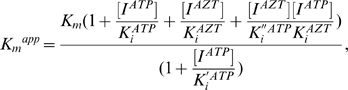
(6)where *K_i_^ATP^* is the dissociation constant of ATP; *K_i_^AZTTP^* is the dissociation constant of AZTTP; [*I^ATP^*] is the ATP concentration; and [*I^AZTTP^*] is the AZTTP concentration.

### Structural Analysis

We constructed the 3-D models of HIV-1 RTs by homology modeling [19] using the Molecular Operating Environment, MOE (Chemical Computing Group, Canada) as previously described [34]. We generated models of the 93JPNH-1 RT and ERT-mt6 RT structures at the pre- and post-translocation stages, which theoretically are competent for the binding of the incoming-ATP. We used two crystal structures of the HIV-1 RTs (PDB code: 1N6Q [14] and 1RTD [1]) as modeling templates. The sequence identities of the 1N6Q and 1RTD with the 93JPNH-1 RT and ERT-mt6 RT are ∼90%. We optimized the 3-D structure thermodynamically by energy minimization using MOE and an AMBER94 force field. We further refined the physically unacceptable local structure of the models on the basis of evaluation by the Ramachandran plot using MOE. The optimized models were docked with ATP with the automated ligand docking program ASEDock2005 [22] (Ryoka Systems, Japan) operated in the Molecular Operating Environment. The RT-template-primer-ATP complex structures were thermodynamically and sterically optimized as described above.

### Site-Directed Mutagenesis

Site-directed mutagenesis was performed with a QuikChange Multi Site-Directed Mutagenesis Kit (Stratagene, USA), using pQE70 (Qiagen, Germany) containing the coding sequence of the p66 subunit of ERT-mt6 as the template. The positions of the amino acid substitutions corresponded to the positions 72, 110, 113, 116, and 219 of 93JP-NH1. The mutations and oligonucleotides used in the mutagenesis reaction were R72A (5′-CGGCCAGCA TTAAATGGgcGAAATTAGTAGATTTCAGAGAG-3′), R72Q (5′-CGGCCAGCATTAAATGGcaGAAATTAGTAGATTTCAGAGAG-3′), D110A (5′-GAAAAAATCAGTAACAGTACTAGcTGTGGGAGATGCATATTTTTC-3′), D110N (5′-GAAAAAATCAGTAACAGTACTAaATGTGGGAGATGCATATTTTTC-3′), D113A (5′-CAGTACTAGATGTGGGAGcTGCATATTTTTCAGTTCCTT-3′), D113N (5′-CAGTACTAGATGTGGGAaaTGCATATTTTTCAGTTCCTT-3′), F116A (5′-GGAACTGAAgcATATGCATCTCCCACATCTAGTACTG-3′), F116L (5′-GGAACTGAcAAATATGCATCTCCCACATCTAGTACTG-3′), K219A (5′-GGGATTTTATACACCAGACgcAAAGCATCAGAAGGAACCTC-3′), and K230(219)Q (5′-GGGATTTTATACACCAGACcAAAAGCATCAGAAGGAACCTC-3′), where the introduced mutations appear in lowercase letters. In all cases, the nucleotide sequences of the complete p66 coding region and of cloning sites were verified with an automated sequencer. The mutant p66 subunits were expressed in XL1-blue and used to form the p51/p66 heterodimer using the p51 subunit of 93JP-NH1 in binding buffer, as described above. The p51/p66 heterodimers were purified by Ni^2+^ affinity chromatography. About 104 to 221 µg of the p51/p66 heterodimers, with about 90% purity as judged by SDS-polyacrylamide gel electrophoresis (Figure S4A), were obtained from a 20 ml culture. The purified RTs were dissolved in the RT stock buffer and kept at −30°C until use.

## Supporting Information

Figure S1 Data on RTs of 93JP-NH1 and ERT-mt6. A. Electrophoresis of the purified p51/p66 heterodimers of HIV-1 RTs. The purified p51/p66 heterodimers of 93JP-NH1 RT (NH1) and ERT-mt6 RT (mt6) were electrophoresed on an SDS-4/20% polyacrylamide gradient gel. The gel was stained with GelCode Blue Stain Reagent (Pierce, USA). (Lanes 1 and 4) Molecular size markers. B. The substrate-velocity curves of purified HIV-1 RTs. RNA-dependent DNA polymerase activity at the indicated concentrations of [α-^32^P]dTTP was measured using purified RTs of 93JP-NH1 (1 nM) and ERT-mt6 (10 nM).
(0.29 MB TIF)

Figure S2 Lineweaver-Burk double-reciprocal plots of AZTTP-dependent inhibition of dTTP incorporation. A. 93JP-NH1 RT. B. ERT-mt6. The initial velocities of dTMP incorporation into poly (rA)⋅p(dT)_12-18_ were measured using [α-^32^P]dTTP and purified RTs in the presence of AZTTP. Reciprocal values of the initial velocities and substrate concentrations are plotted.
(0.15 MB TIF)

Figure S3 Docking simulations of ATP with RT-template-primer ternary complex models. A and C: 93JP-NH1 RT. B and D: ERT-mt6 RT. The 3-D models of the p66-template-primer complexes at the pre-translation stage (A and B) and the post-translation stage (C and D) were constructed by a homology modeling technique and docked with ATP using the ASEDock2005 (see Materials and Methods). Catalytic clefts composed of fingers, palm, and thumb subdomains are shown. ATP, red sticks; p66 main chain, grey ribbon; template-primer, grey sticks; motif A, blue ribbon.
(1.93 MB TIF)

Figure S4 Data on RT mutants from the ERT-mt6 RT. A. Electrophoresis of the purified RT mutants from the ERT-mt6 RT. B. dTMP incorporations into poly (rA)⋅p(dT)_12-18_ by the mutant RTs. RNA-dependent DNA polymerase activity of the purified RTs (20 nM) was measured using a [α-^32^P]dTTP and poly (rA)⋅p(dT)_12-18_ system. C. Fold increases in the IC_50_ of AZTTP by ATP addition. IC_50_ values of AZTTP with RT mutants were calculated from the amounts of [α-^32^P]dTTP incorporation in the presence of various concentrations (0–1 µM) of AZTTP and 5 mM ATP. Fold increases in IC_50_ compared to the values without ATP are shown. D. The substrate-velocity curves of purified HIV-1 RTs in the presence of ATP. RNA-dependent DNA polymerase activity of the purified mutant RTs was measured using various concentrations of [α-^32^P]dTTP and poly (rA)⋅p(dT)_12-18_ in the presence of ATP. Representative results with D113A RT (left) and K219A RT (right) are shown.
(0.39 MB TIF)
