# Supplementary figures and images for: Allosteric Regulation of HIV-1 Reverse Transcriptase by ATP for Nucleotide Selection
Source: PLoS One. 2010 Jan 25;5(1):e8867. doi: 10.1371/journal.pone.0008867 (PMC2810339; doi:10.1371/journal.pone.0008867)

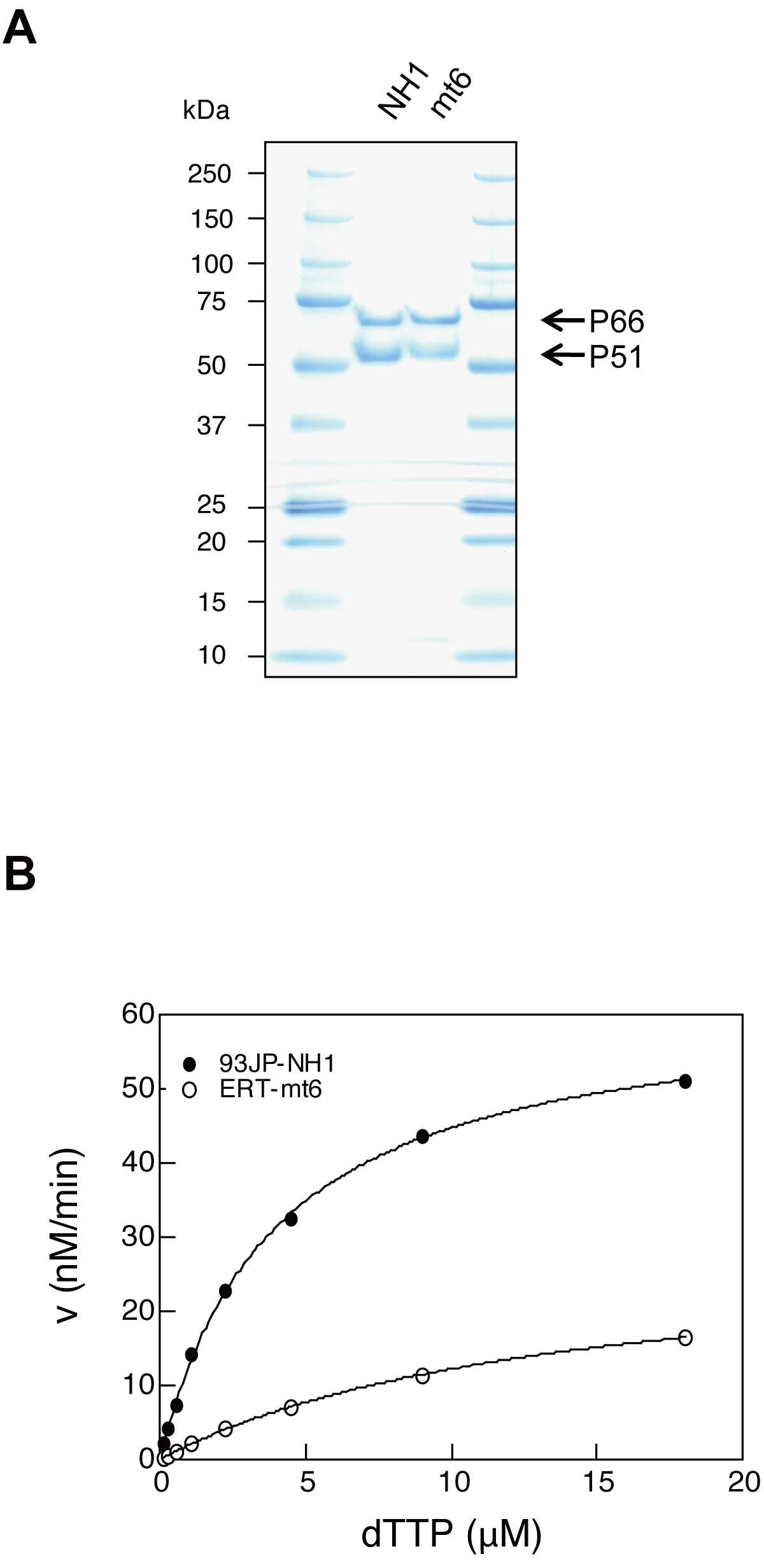

Supplement: Figure S1 — Data on RTs of 93JP-NH1 and ERT-mt6. A. Electrophoresis of the purified p51/p66 heterodimers of HIV-1 RTs. The purified p51/p66 heterodimers of 93JP-NH1 RT (NH1) and ERT-mt6 RT (mt6) were electrophoresed on an SDS-4/20% polyacrylamide gradient gel. The gel was stained with GelCode Blue Stain Reagent (Pierce, USA). (Lanes 1 and 4) Molecular size markers. B. The substrate-velocity curves of purified HIV-1 RTs. RNA-dependent DNA polymerase activity at the indicated concentrations of [α-32P]dTTP was measured using purified RTs of 93JP-NH1 (1 nM) and ERT-mt6 (10 nM). (0.29 MB TIF) [file pone.0008867.s001.tif]

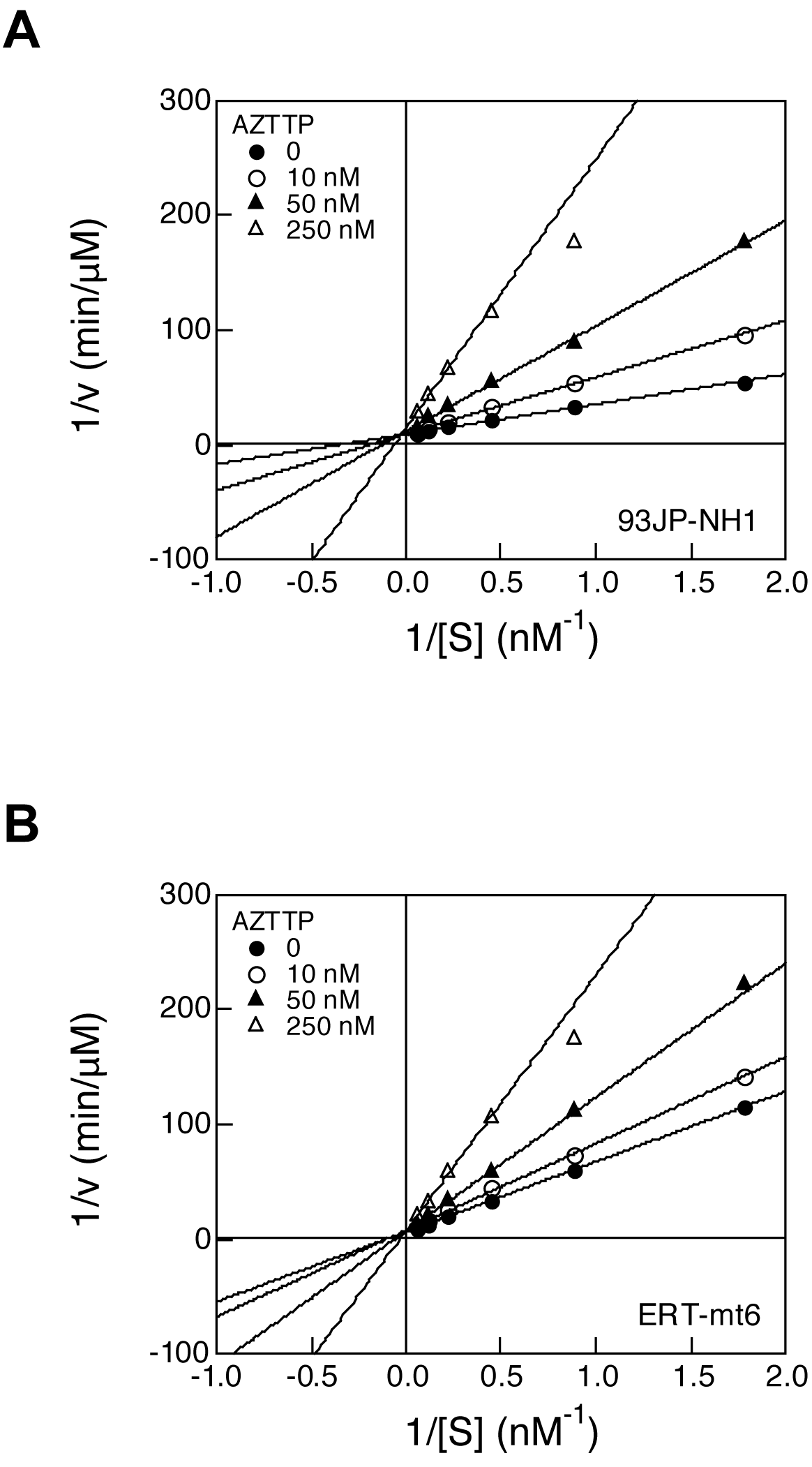

Supplement: Figure S2 — Lineweaver-Burk double-reciprocal plots of AZTTP-dependent inhibition of dTTP incorporation. A. 93JP-NH1 RT. B. ERT-mt6. The initial velocities of dTMP incorporation into poly (rA)⋅p(dT)12-18 were measured using [α-32P]dTTP and purified RTs in the presence of AZTTP. Reciprocal values of the initial velocities and substrate concentrations are plotted. (0.15 MB TIF) [file pone.0008867.s002.tif]

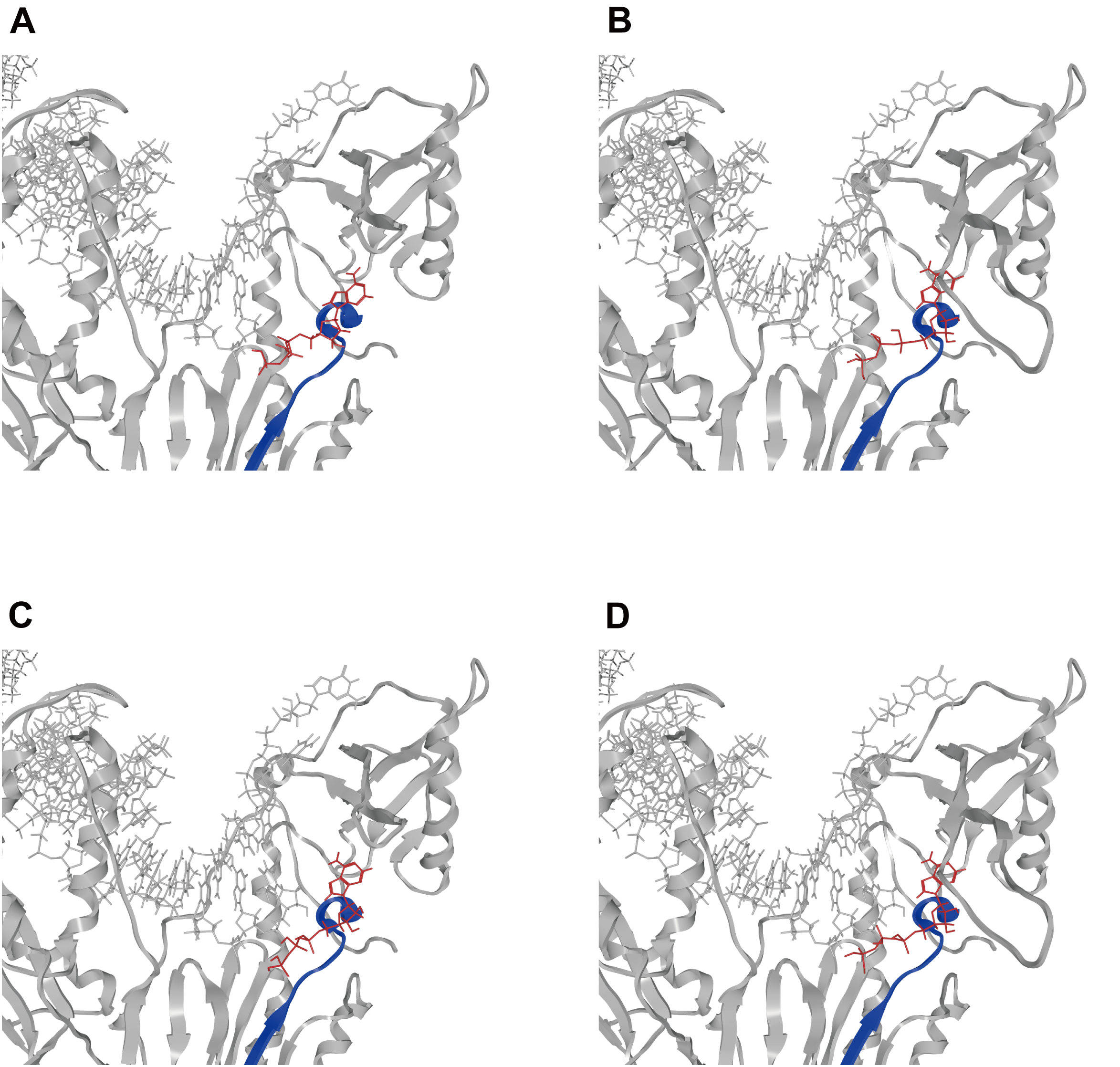

Supplement: Figure S3 — Docking simulations of ATP with RT-template-primer ternary complex models. A and C: 93JP-NH1 RT. B and D: ERT-mt6 RT. The 3-D models of the p66-template-primer complexes at the pre-translation stage (A and B) and the post-translation stage (C and D) were constructed by a homology modeling technique and docked with ATP using the ASEDock2005 (see Materials and Methods). Catalytic clefts composed of fingers, palm, and thumb subdomains are shown. ATP, red sticks; p66 main chain, grey ribbon; template-primer, grey sticks; motif A, blue ribbon. (1.93 MB TIF) [file pone.0008867.s003.tif]

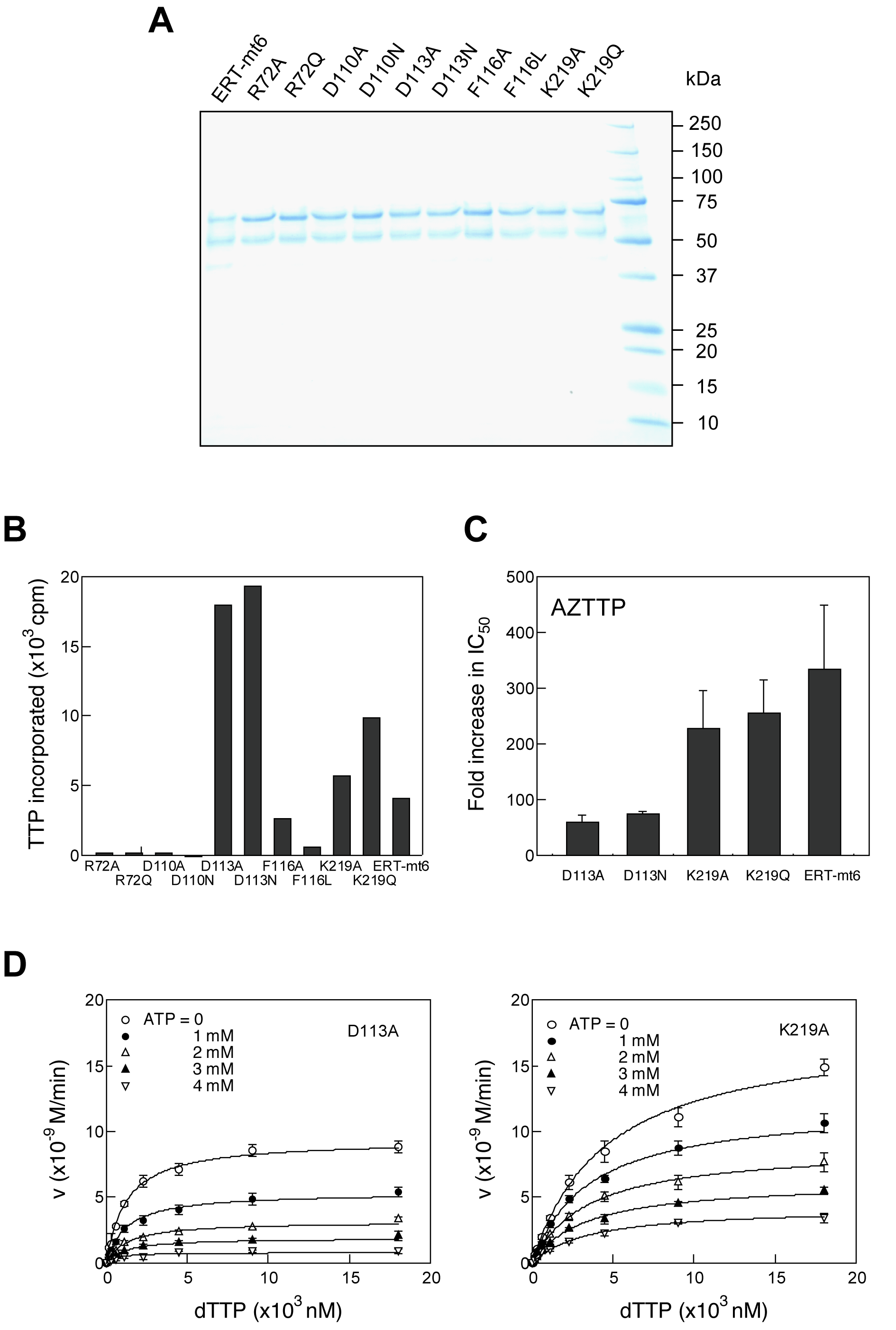

Supplement: Figure S4 — Data on RT mutants from the ERT-mt6 RT. A. Electrophoresis of the purified RT mutants from the ERT-mt6 RT. B. dTMP incorporations into poly (rA)⋅p(dT)12-18 by the mutant RTs. RNA-dependent DNA polymerase activity of the purified RTs (20 nM) was measured using a [α-32P]dTTP and poly (rA)⋅p(dT)12-18 system. C. Fold increases in the IC50 of AZTTP by ATP addition. IC50 values of AZTTP with RT mutants were calculated from the amounts of [α-32P]dTTP incorporation in the presence of various concentrations (0–1 µM) of AZTTP and 5 mM ATP. Fold increases in IC50 compared to the values without ATP are shown. D. The substrate-velocity curves of purified HIV-1 RTs in the presence of ATP. RNA-dependent DNA polymerase activity of the purified mutant RTs was measured using various concentrations of [α-32P]dTTP and poly (rA)⋅p(dT)12-18 in the presence of ATP. Representative results with D113A RT (left) and K219A RT (right) are shown. (0.39 MB TIF) [file pone.0008867.s004.tif]
